# Voltage-Dependent Interaction of Capsaicine and Protons on TRPV1-Receptors

**Published:** 2017

**Authors:** E.A. Tsvetkov, N.N. Potatieva, K.V. Bolshakov

**Affiliations:** Sechenov Institute of Evolutionary Physiology and Biochemistry of the Russian Academy of Sciences (IEPhB RAS), pr. Torez 44, St. Petersburg, 194223, Russia; Federal State Budgetary Educational Institution of Higher Professional Education «Saint-Petersburg State University», Universitetskaya nab. 7–9, St. Petersburg, 199034, Russia

**Keywords:** TRPV1, Capsaicin, pH, Agonists of TRPV1-receptors, Interaction of agonists of TRPV1-receptors

## Abstract

The interaction of TRPV1-receptors agonists (capsaicin and protons) has been studied on cultured CHO cells transfected by TRPV1-receptors. Using the whole-cell patch-clamp approach, it was shown that summation of the currents induced by agonist application was dependent on the membrane potential. The TRPV1-mediated currents induced by the pH and Capsaicin demonstrated arithmetical summation at potentials between 40–-40 mV, while they were potentiated at potentials below -40 mV. Currents induced by the pH and Capsaicin combined were higher in comparison with the arithmetic sum of the currents induced by the pH and Capsaicin applied separately at such potentials. Such a potential dependence seems to be a base of the sensitization that is induced by inflammation or pain, when concentrations of proinflammatory mediators acting as TRPV1 agonists are increasing. Further depolarization induced by TRPV1 activation doesn’t generate potentiation, which might serve as a protective mechanism to restrict their activity.

## INTRODUCTION

Capsaicin receptors (TRPV1) are complexly organized polymodal sensory systems that react to a variety of stimuli of both chemical and physical nature [1-12]. In most cases, these stimuli cause the opening of a pore of the channel-receptor complex and elicit a transmembrane ion current.

The polymodality of TRPV receptors allows them to react not only to the application of individual agonists, but also to their combinations. The latter generally causes a mutual potentiation of responses, and this phenomenon has been previously described for various combinations of agonists, including capsaicin, arachidonic acid derivatives, pH, as well as physical stimuli such as changes in temperature, membrane potential or pressure [1-13]. In particular, the data show that extracellular acidification of the environment increases TRPV1 receptors’ sensitivity to capsaicin [4, 14, 15], while an increase in temperature shifts their activation by potential toward depolarization [16].

Since acidification of the environment is an important sign of a developing inflammatory response [17], potentiation of TRPV1 receptors, when combined with the effect of other agonists (e.g. capsaicin), can be considered as part of the signaling mechanism triggered in a cell in response to inflammation. Elucidation of the phenomenology of such potentiation and its mechanism are of practical interest both for understanding the inflammatory process itself and for studying ways to attenuate it.

Nevertheless, an analysis of the published data reveals that understanding of the potentiation of TRPV1 receptors, observed after their simultaneous activation by two agonists, is incomplete. In particular, there are no data on whenever this interaction may depend on the membrane potential, which is an important parameter of a cell that affects both the signaling cascades and receptors themselves, including capsaicin receptors.

The aim of this work was to study the interaction of capsaicin and pH at different membrane potentials.

It has been demonstrated that nonlinear summation of TRPV1 receptor responses to combined exposure to protons and capsaicin is observed only at potentials close to the resting potential. When the membrane is depolarized due to the development of an inflammation or various pathologies, the summation becomes linear. This property of TRPV1 receptors seems to be protective, limiting their hyperactivation in pathological conditions.

## EXPERIMENTAL PART

The work was performed on recombinant TRPV1 receptors constitutively or transiently expressed in CHO cells. CHO cells were cultured under standard conditions in a DMEM/F12 medium (Dulbecco’s modified Eagle’s medium, Biolot) with 10% fetal bovine serum (Hyclone) and 1% gentamycin in a humidified incubator, at 5% CO_2_ and 37°C. Transfection was performed with lipofectamine-2000 (Invitrogen, USA) according to the manufacturer’s recommendations. For transfection, 0.5 μg of the plasmid encoding TRPV1 and 0.5 μg of the plasmid encoding the eGFP gene were added to a 35-mm Petri dish with the CHO culture. The plasmids were provided by Dr. Staruschenko and Dr. Medina, respectively. The experiments were performed on Days 2 to 5 after the transfection. The transfection efficiency was assessed by the fluorescence intensity of GFP, as measured by a MF-51 microscope. Part of the work was performed on constitutively transfected CHO cells, kindly provided by E.V. Grishin. There were no differences between the two types of transfection: therefore, the data were combined.

Agonist-evoked currents were recorded at different membrane potentials in the voltage clamp “whole cell” mode. The EPC10 amplifier (HEKA Electronik, USA) and the PatchMaster v8.2 software package (HEKA Elektronik) were used in the study. The test solutions were applied using a NANION solution exchange system (Nanion, Germany) through a micromanifold with an internal diameter of 250 μm; the time for replacing the solution was about 100 ms. To reduce the desensitization of the receptors due to repeated application of solutions, the frequency of their application did not exceed 1 time in 45 s. The recording pipettes were prepared on a P-87 microfuge (Sutter Instruments Co., USA) from borosilicate capillaries with a filament (Sutter Instruments Co.). The outer and inner diameters of the capillaries were 1.5 and 0.86 mm, respectively. The resistance of the filled pipettes was 3–6 MΩ. For electrophysiological tests, the cells were transferred to a solution with the following composition (mM): 140 NaCl, 5 KCl, 1 MgSO_4_, 2.5 CaCl_2_; 10 glucose; 10 HEPES, pH 7.4. The composition of the pipette solution (mM): 100 CsF, 40 CsCl, 5 NaCl, 0.5 EGTA, 10 HEPES, pH 7.2. Reagents from Sigma (USA) were used for the preparation of the solutions. Capsaicin was diluted according to the recommendations of the manufacturer (Sigma), in 96% ethanol to a concentration of 10 mM, and the required amount was added to obtain the indicated final concentrations.

Statistical processing of the data was performed in EXCEL. The comparison of mean values and assessment of their placement into one/different sets was carried out using the paired Student t-test, since the sets of means were obtained on the same cell. Since different cells had different amplitudes of responses to capsaicin and protons (due to differences in receptor density and cell size), for measurement of the potentiating effect we normalized the amplitudes of the current of each cell by the amplitude of the capsaicin response of that cell. The EC_50_ and Hill coefficient were estimated using the ORIGIN package with approximation of the experimental data to the theoretical curve by Hill’s equation: *I *= *I_max_*(1/(1+ (EC_50_/[*C*])*s*)), where *Imax *is the current amplitude at the saturating concentrations of the ligands, capsaicin or pH; *I *is the current amplitude at the current ligand concentration [*C*]; EC_50_ is the concentration of the half-maximum effect; and *s *is the Hill coefficient. EC_50_ for proton concentration is indicated in the text in units of acidity, pH_50_.

## RESULTS AND DISCUSSION

**Fig. 1:**
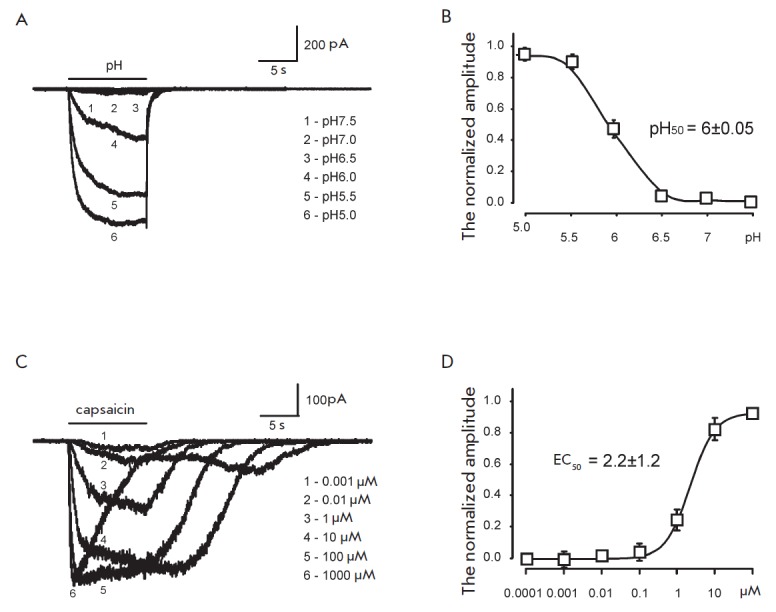
The sensitivity of TRPV1 receptors to capsaicin and pH. A: The responses of a representative cell elicited by the application of a solution with a different pH. B: The dose-response curve, normalized to the amplitude of the current elicited by a solution with pH5.0; pH_50_ = 6.0 ± 0.05; n = 10. C: The responses of a representative cell elicited by the application of a solution with a different concentration of capsaicin. D: The dose-response curve, normalized by the amplitude of the current elicited in a solution at a saturated concentration of capsaicin; EC_50_ = 2.2 ± 1.2 μM; n = 10.

To study the combined effect of the TRPV1 receptor agonists, we first assessed the range of the working concentration for the application of each agonist alone. For this purpose, dose-response curves were obtained for capsaicin and pH at a membrane potential of -80 mV. The results are shown in *Fig. 1*. The data show that responses to acidification of the environment start to appear at pH 6.5, and their amplitude subsequently increases with increasing acidity and reached saturation at pH 5.5 and above. Responses to capsaicin started to appear at a concentration of 0.01 μM and reached saturation at values close to 100 μM. However, as the concentration of capsaicin increased, the amplitude of the responses decreased. The drop in the response amplitude at high concentrations of capsaicin may be associated with its nonspecific action on the cell membrane. Therefore, concentrations of capsaicin above 100 μM were not used in the subsequent experiments. EC_50_ calculated from these experiments for capsaicin was 2.2 ± 1.2 μM (*n *= 10), and pH50 for TRPV1 receptors was 6.0 ± 0.05 (n = 10), which agrees well with the published data [4]. It should be noted that neither capsaicin nor pH in the studied concentrations elicited a current in non-transfected CHO cells. Responses to capsaicin and pH in transfected cells were blocked by 10 μM ruthenium red. This indicates that the currents recorded under these conditions are mediated by the TRPV1 receptors.

**Fig. 2:**
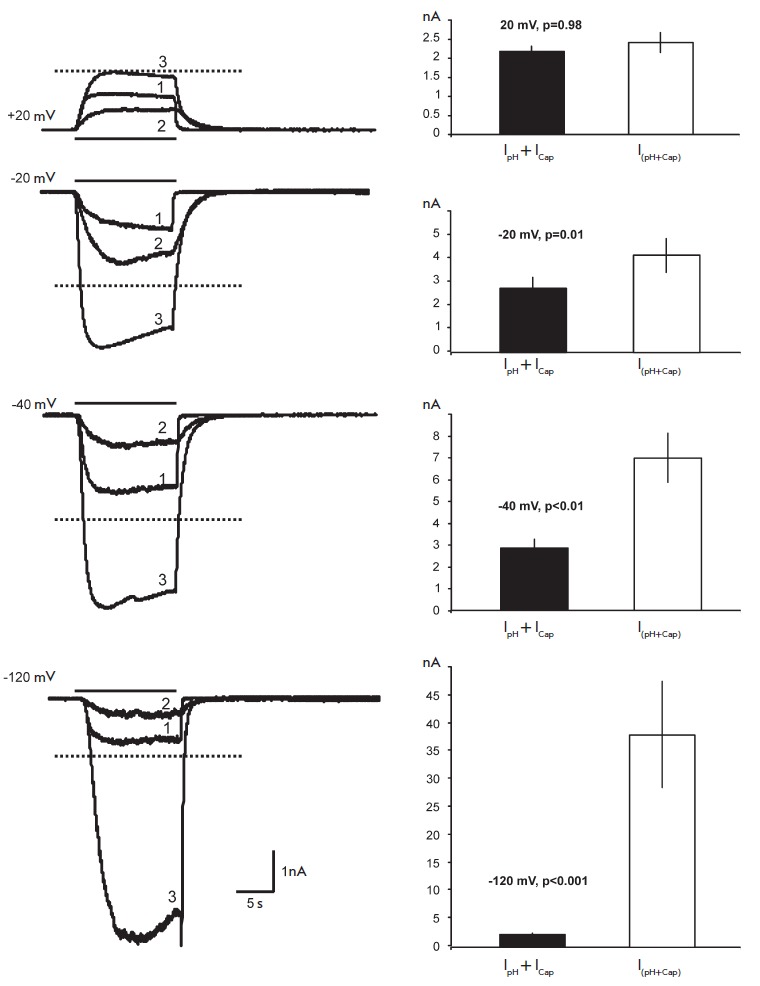
The interaction of pH and capsaicin at different holding potentials. Left column: 1 – currents elicited by the application of a solution with pH5.0. 2 – currents elicited by the application of a solution with 0.1 μM of capsaicin. 3 – currents elicited by the application of a solution with pH5.0 and 0.1 μM of capsaicin given together. The dotted line is a theoretical value of the arithmetical sum of the current amplitudes elicited by the application of a solution with pH 5.0 and 0.1 μM of capsaicin, respectively. Right column: comparison of the theoretical and empirical sums of the currents elicited by a combined application of pH and capsaicin.

The following protocol was used to study the interactions of the proton and capsaicin effects at different potentials. First, we recorded the response to the application of a solution with a certain pH, then the response to a solution of capsaicin at a certain concentration; then, we applied the solution with both the pH and capsaicin concentrations as described above. In a separate series of experiments, we showed that a change in the order (sequence) of the application of agonists did not affect the amplitudes of the responses. The data analysis included a comparison of the response amplitude for a combined application of capsaicin and protons (*I*_(pH+Cap)_) with the sum of the response amplitudes (*I*_pH_ + *I*_Cap_) obtained when capsaicin (*I*_Cap_) and proton (*I*_pH_) were applied separately. The obtained data are shown in *Fig. 2*, where 0.1 μM and pH 5.0 were taken as the test concentrations of capsaicin and protons, respectively. *Fig. 2*demonstrates that the amplitude of the responses to the combined application of the agonists used (*I*_(pH+Cap)_) can significantly exceed the sum of the response amplitudes (*I*_pH_ + *I*_Cap_) obtained when capsaicin (*I*_Cap_) and pH (*I*_pH_) are applied separately. This potentiation depends on the level of the membrane potential and is most pronounced under conditions of hyperpolarization of the cell. At a potential of -40 mV, the current amplitude caused by 0.1 μM capsaicin at pH 5.0 significantly exceeded the sum of the responses caused by the application of 0.1 μM capsaicin, followed by the lowering of pH to 5.0 (p < 0.01); at a potential of -120 mV, this difference was significant at p < 0.001. Displacement of the MP towards the depolarization lowers the value of potentiation and brings the amplitude of the response for a combined application of capsaicin and protons closer to the sum of the responses for their individual application. At 20 and -20 mV, the differences between the amplitudes of the response to a combined treatment and the sum of the amplitudes of the individual responses to agonists are unreliable.

**Fig. 3:**
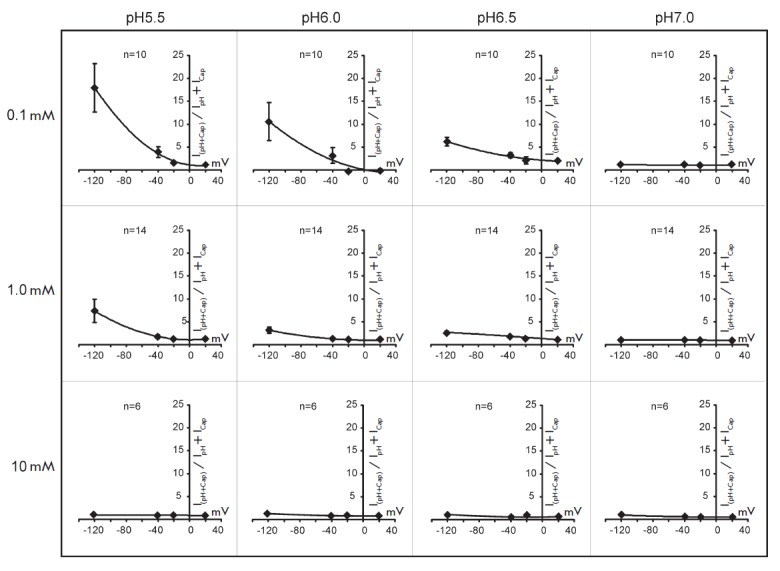
The dependence of capsaicin receptors potentiation on the membrane potential at different pH and capsaicin concentrations. Explanation of the experimental protocol and the analysis procedure are given in the text.

For a more detailed characterization of the phenomenon of TRPV1 receptors potentiation, similar experiments were repeated at different concentrations of the agonists. The range of capsaicin concentration varied from 0.1 to 10 μM; and pH levels, from 5.5 to 7.0. The ratio of the amplitude of the current elicited after a combined application of the agonists (*I*_(pH+Cap)_) to the sum of the amplitudes of the currents elicited by an individual application of these agonists (*I*_pH_ + *I*_Cap_) was used as a parameter for evaluating the potentiation. The values of (*I*_(pH + Cap)_)/(*I*_pH_ + *I*_Cap_) are presented in *Fig. 3* in graphic form. In this figure, the columns present the data obtained at the same pH values, where the concentration of capsaicin was varied, while the rows present the data obtained at the same concentrations of capsaicin, where the pH was varied. It should be noted that the potentiation effect was not observed at a capsaicin concentration greater than 10 μM; therefore, no experiments with a higher concentration of the agonist were performed.

*Fig. 3* shows that the potentiation effect depends on all the parameters controlled in these tests. The greatest effect was observed under conditions of maximum cell hyperpolarization with maximum acidification of the environment and the lowest concentrations of capsaicin (see upper left corner of the table in *Fig. 3*). It is clear that the extent of potentiation of TRPV1 receptors directly depends on the concentration of protons and increases with acidification at a constant concentration of capsaicin. The potentiating effect of pH better manifests itself at low concentrations of capsaicin and practically disappears when the concentration reaches 10 μM. Thus, there is an inverse relationship between the potentiation of the pH-response and the capsaicin concentration, and an increase in the concentration of capsaicin results in a decrease in the potentiation.

**Fig. 4:**
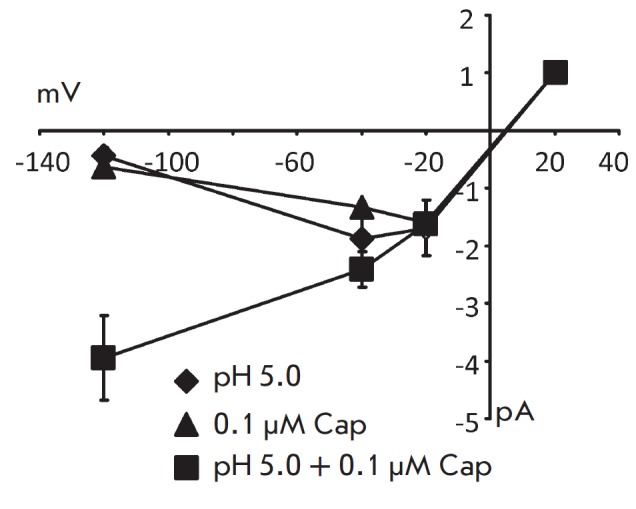
I/V relationship of TRPV1-receptor responses elicited by the application of agonist. Triangles – I/V relationship of TRPV1-receptor responses elicited by the application of a solution with 0.1 μM of capsaicin. Rhombs – I/V relationship of the TRPV1-receptor responses elicited by the application of a solution with pH5.0. Rectangles – I/V relationship of the TRPV1-receptor responses elicited by the application of a solution with pH5.0. and 0.1 μM capsaicin given together.

The sensitivity of the potentiation of capsaicin receptors to the membrane potential of the cell suggests that the application of capsaicin in conditions of lower pH of the environment would lead to a change in the current-voltage relationship of the capsaicin receptor responses to the action of the agonists. To verify this assumption, we compared the current-voltage relationship of the channels obtained by activating the receptors with capsaicin, pH, and the combined application of these agents at concentrations which corresponded to the maximum value of the potentiation effect in the previous experiments. The result of these experiments is shown in *Fig. 4*. The responses to pH and capsaicin are characterized by inward rectification, which agrees well with the published data [2, 14]. In the case of a combined application of protons and capsaicin, the degree of rectification decreases. The weakening of the rectifying properties of TRPV1 receptors can be considered as an element of the mechanism that regulates the signaling functions of the receptors during the development of inflammatory reactions, pain, thermoregulation, and other functions in which TRPV1 receptors are involved [1-12].

The data obtained is insufficient to draw a definite conclusion on the mechanisms of potentiation of capsaicin receptors in the case of a combined application of capsaicin and protons. However, considering the change in the rectifying properties of the channel observed when the agonists are applied together, the potential mechanism of this phenomenon can be both a voltage-dependent increase in the sensitivity of these receptors to one of the agonists in the presence of the other and a modification of the parameters of receptor inactivation under these experimental conditions. Verification of these assumptions and identification of the mechanisms of interaction between the responses caused by the activation of the receptors by protons and capsaicin at different potentials, as well as the elucidation of the physiological significance of this interaction, requires further studies.

## CONCLUSION

The identified relationships between the potentiating action of TRPV1 agonists when they are applied together and the membrane potential reveals another feature of TRPV1 receptors that allows them to fine-tune their response to a combination of external and internal factors. For example, the potentiation of TRPV1 responses under hyperpolarization conditions enables the involvement of these receptors in an early stage of inflammation, when the concentration of inflammatory agents is not yet too high. Since the triggering of these receptors can be associated with the initiation of apopoptosis, the disappearance of a response potentiation to a combined application of the agonists under conditions of depolarization will serve as a protective mechanism. However, understanding of the functional significance of the amplitude of TRPV1 responses, as well as the elucidation of the molecular mechanisms that mediate the interaction of the different agonists of these receptors, requires further research.

## Abbreviations

CHO: Chinese Hamster Ovary (cells of connective tissue of Chinese hamster ovary)
TRPV1: Transient Receptor Potential channel subfamily V member 1 (vanilloid receptor, type 1)
GFP: Green Fluorescent Protein
MP: membrane potential
